# P065. Cluster headache: when to worry?

**DOI:** 10.1186/1129-2377-16-S1-A101

**Published:** 2015-09-28

**Authors:** Matteo Bellamio, Giulia Toldo, Federico Mainardi, Giorgio Zanchin, Ferdinando Maggioni

**Affiliations:** Headache Centre, Department of Neurosciences, University of Padua, Padua, Italy; Headache Centre, Neurological Division, SS Giovanni e Paolo Hospital, Venice, Italy

## Introduction

Cluster headache (CH) is a rare and disabling primary trigeminal autonomic cephalalgia which interests especially the male population with smoking and alcohol ingestion habits.

CH typically occurs at the same time of the day, once to eight times per day, and in the same period of the year. CH is featured by severe unilateral peri-orbital and/or temporal pain lasting from 15 to 180 minutes if untreated, associated with at least one autonomic symptom (conjunctival injection, lacrimation, nasal congestion, rhinorrhea, facial sweating, miosis, ptosis and eyelid edema)[1]. CH secondary forms can be a manifestation of neurological pathologies such as brain tumor, cerebral venous thrombosis[2], arterial aneurisma or multiple sclerosis[3].

## Case report

A 29-year-old man with silent medical history came to our observation on June 10^th^. Last December he experienced his first episode of headache described as right-sided pulsating pain, in fronto-orbital and nasal regions, which lasted two hours and recurred twice a day around 2.00 am and 2.00 pm; they were accompanied by ipsilateral conjuntival injection, lacrimation and rhinorrea. This episode ended just a few days later. At the end of April he reported a second episode with the same features but, after a week, the pain became more intense, pressing and localized all over the head associated with transient fluctuating reduction of visual acuity. He performed brain MRI which showed nonspecific white matter lesions localized in periventricular areas. He went to the Emergency Department presenting fever, signs of meningeal irritation and endocranial hypertension. During the hospitalization an MRI with vascular assessment was performed; it confirmed the white matter lesions and found a thrombosis in the left external jugular vein which climbed up to the distal portion of sigmoid sinus. Cerebrospinal fluid (CSF) isoelectric focusing after lumbar puncture was positive for oligoclonal bands in the gamma regions, while cytological, biochemical, serological, and virological findings of the CSF and blood were normal.

## Discussion

According to 2013 ICHD-III criteria, our patient suffered from episodic CH. In the literature we found data on cluster-like headache which can be the only manifestation of a secondary and more dangerous pathology. In our case both CH periods were not involved in the pathogenesis of thrombosis or CSF alterations. It is important to pay attention if there are any changes in headache in the patient's history because they could be a warning of a secondary form.

Written informed consent to publication was obtained from the patient(s).
